# Solid Waste Management Solutions for a Rapidly Urbanizing Area in Thailand: Recommendations Based on Stakeholder Input

**DOI:** 10.3390/ijerph15071302

**Published:** 2018-06-21

**Authors:** Nachalida Yukalang, Beverley Clarke, Kirstin Ross

**Affiliations:** 1 College of Science and Engineering, Flinders University, Bedford Park, Adelaide, SA 5042, Australia; kirstin.ross@flinders.edu.au; 2 College of Humanities, Arts and Social Sciences, Flinders University, Bedford Park, Adelaide, SA 5042, Australia; beverley.clarke@flinders.edu.au

**Keywords:** integrated solid waste management, municipal solid waste management, opinions, solutions, urbanizing, developing countries

## Abstract

Municipal solid waste is a significant problem, particularly in developing countries that lack sufficient infrastructure and useable land mass to process it in an appropriate manner. Some developing nations are experiencing a combination of issues that prevent proper management of solid waste. This paper reviews the management of municipal solid waste in northeast Thailand, using the Tha Khon Yang Sub-district Municipality (TKYSM) in Maha Sarakham Province as a case study. The combination of rapid population and economic growth and its associated affluence has led to an increase in the use of consumer items and a concomitant increase in the production of municipal solid waste. In the TKYSM there is pressure on local government to establish a suitable waste management program to resolve the escalating waste crisis. The aim of this study is to provide viable solutions to waste management challenges in the TKYSM, and potentially to offer guidance to other similar localities also facing the same challenges. It is well established that successful changes to waste management require an understanding of local context and consideration of specific issues within a region. Therefore, extensive community consultation and engagement with local experts was undertaken to develop an understanding of the particular waste management challenges of the TKYSM. Research methods included observations, one-on-one interviews and focus groups with a range of different stakeholders. The outcomes of this research highlight a number of opportunities to improve local infrastructure and operational capacity around solid waste management. Waste management in rural and urban areas needs to be approached differently. Solutions include: development of appropriate policy and implementation plans (based around the recommendations of this paper); reduction of the volume of waste going to landfill by establishing a waste separation system; initiation of a collection service that supports waste separation at source; educating the citizens of the municipality; and the local government staff, and for the local government to seek external support from the local temples and expertise from the nearby university.

## 1. Introduction

Globally, population growth, together with economic growth and associated consumption behaviour, has resulted in a significant increase in solid waste production [1,2]. In developing countries, managing municipal solid waste (MSW) is a serious problem [2,3]. Urbanisation and increasing affluence have resulted in a significant increase in volumes of discarded materials [4,5,6]. The problem of MSW is particularly challenging for large cities in developing countries [7,8] and for local levels of government that are typically responsible for managing waste [3,9].

MSW is defined as local waste generated by households and commercial and governmental enterprises. It includes packaging, food waste, grass clippings, clothing, paper and other solid forms of waste, but does not include hazardous and infectious waste or sewage [3,9,10]. It is estimated that the volume of MSW could double from current levels of 1.3 billion tonnes annually to 2.6 billion tonnes by 2025 [10].

In developed countries, MSW is usually well managed. Often the highly technical and complicated methods of dealing with MSW used effectively in developed countries are brought to developing countries. However, these solutions are often not sustainable due to lack of capacity and the incongruity of trying to establish systems in dissimilar situations [11,12].

Municipal solid waste management (MSWM) in developing countries accounts for between 20% and 50% of local government budgets [3]. Studies show that more than 50% of developing countries’ populations lack consistent access to waste collection services [5,6]. Disposal methods often include open dumping and open burning [5]. The World Bank reports vast amounts of uncollected waste in urban areas; estimates suggest between 40% and 70% of discarded materials remaining uncollected [6]. This pollution leads to significant impacts on human health and the environment [4,13].

In keeping with global trends, waste generation in Thailand is increasing. From 2008 to 2016, waste generation increased from around 24 million tonnes per year in 2008 to over 27 million tonnes [14,15]. The Thai Government in recognising the problem released its Roadmap for Municipal and Hazardous Waste in August 2014 [16]. The Roadmap was coupled with a devolution of responsibility for MSWM from central to local government [17]. The central Thai government transferred functions, budget, and personnel to local governments, which means local governments now play a lead role in the management of solid waste within their jurisdictions [18]. There is limited data about the specific amount of waste generated in individual districts Thailand; however, the Pollution Control Department of Thailand reports that the northeast Thailand is generating of highest volume of waste in the country [19].

However, there are many issues preventing the implementation of a successful MSWM system in Thailand. Thai municipal governments are focused on other pressing problems such as water and sewage infrastructure, road maintenance, public amenities, and disaster response. Waste management is often not the priority [3]. In 2016, it was estimated that of the 7777 Thai local governments, only 60% provided a waste collection service. Of this 60%, only two-thirds is transferred to sanitary landfill sites [14]. An absence of a functioning MSWM service leads to open burning and open dumping and environmental pollution and health issues. There have been public protests in Thailand about such insanitary conditions [20].

**Figure 1:**
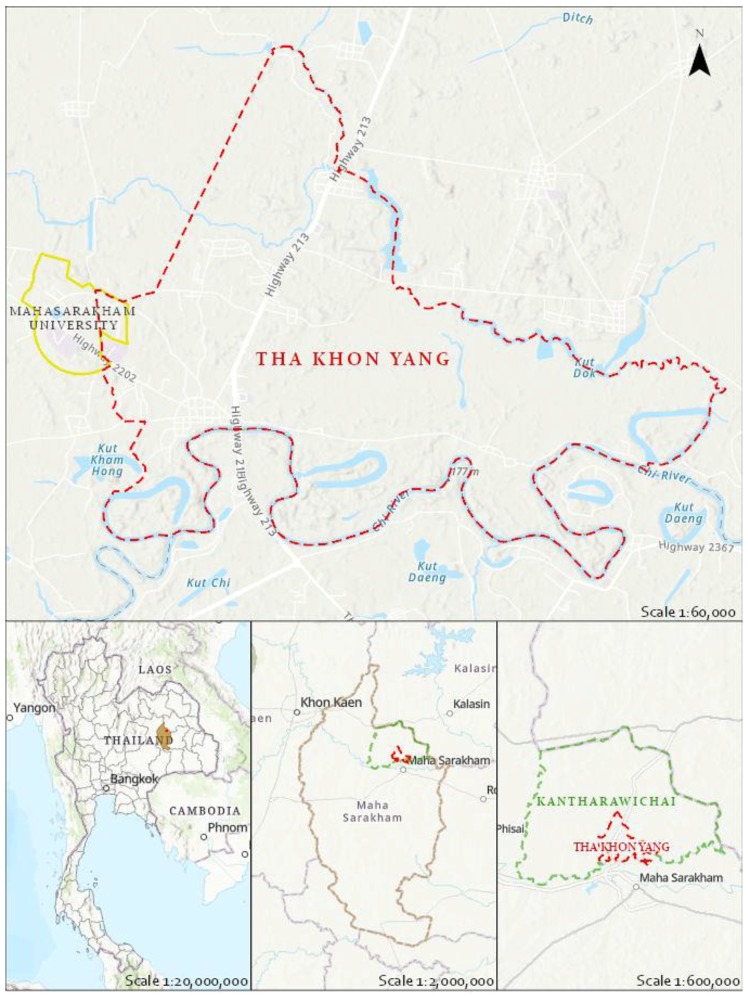
Location of Tha Khon Yang Sub-district in the Maha Sarakham Province in the northeast of Thailand. (Sources: Esri, USGS, NGA, NASA, CGIAR< N. Robinson, NCEAS, NLS, OS, NMA. Geodatastyrelsen, Rijkswaterstaat, GSA, Geoland, FEMA, Intermap and the GIS user community Boundaries: GISTA (Geo-Informatics and Space Technology Development Agency).

This paper focusses on the Tha Khon Yang Sub-district in the Maha Sarakham Province in the northeast of Thailand (Figure 1). Tha Khon Yang (TKY) comprises 15 villages. Maha Sarakham city has become an education hub with several universities and colleges, the largest being the Mahasarakham University. In the last two decades, five of the 15 villages of TKY have transitioned from rural to urban settlements. In the TKY, there are 8400 permanent and approximately 25,000 temporary residents (students or workers) [21]. As a result of the expansion of the Mahasarakham University accommodation and commercial enterprises have spread into the adjacent Khamriang Sub-district as well as the TKY. Urbanisation has had the combined effect of increased consumption of goods and concentrated the waste that is generated. In 2010 administrators of the Mahasarakham University, concerned with the increasing volume of waste produced within its campuses, created a zero-waste policy and implementation plan. This plan included additional waste management facilities and promotion of awareness of solid waste management approaches for their staff and students [22]. The Mahasarakham University introduced recycling banks, waste separation sites, and bio-fertilizer production from compost [23]. This reduced the volume of waste going to landfill by several tonnes per day. In 2015, the University was recognised by the Green University campaign for its commitment by achieving a place in the UI Green Metric World University Ranking [24]. 

The Department of Public Health and Environment in the Tha Khon Yang Sub-district Municipality (TKYSM) has responsibility for municipal solid waste. The Department has no formalised or articulated policy or plan to guide waste management; nor is there a published strategic plan or vision for future waste management. In addition, the TKYSM waste collection team has responsibilities outside of waste management. The TKYSM Department of Public Health and Environment, in addition to MSWM, is responsible for assisting in the delivery of health promotion initiatives, food safety, vector control and emergency response. These functions are carried out by a team of 10 operational and 2–3 managerial staff. 

The annual revenue for the TKY raised from the contribution from the central Thai government, together with fees paid by residents and businesses for waste management is approximately 3 million baht (US$94,800—based on 2015 figures). This revenue funds waste collection (the purchase and maintenance of trucks, fuel, salaries of staff), and landfill fees for final waste disposal. The TKYSM pays a significant proportion of its budget in landfill fees (400 baht (US$11.60) per tonne of waste).

Currently, the TKYSM provides some large ‘community’ bins, while some residents provide their own bins or bags, which are placed randomly on curbsides. There are no fixed waste collection points. Twice daily from Monday to Saturday, three waste collection trucks (each with one driver and two collectors) service seven zones in TKY. This collection service covers 76% of the TKY municipality [25]. Each truck services a different zone. One collects from the off-campus dormitories, the second covers rural areas and the third covers the urban area. Each truck follows its own standard route. The trucks are not always able to complete one collection cycle in a day because trucks, when filled to capacity before their round completion, must go to the landfill site 25 km away. The trucks recommence their collection cycle where they left off. However, in the meantime, waste accumulates throughout the municipality on curbsides, where it is placed continuously for collection because residents have no notification of a fixed collection time. During the collection process, to generate extra money for personal profit, the waste collectors try to select and separate sellable items. Of the estimated 15 tonnes of waste generated [26], only 10 tonnes are collected and transferred to the landfill. The rest remains in situ as pollution.

In a bid to reduce the volume of waste going to landfill, TKYSM encourages households to separate recyclables and compost organic waste and, at times, has encouraged citizens to burn their waste. To date, these approaches have not been successful. The challenges of waste management for TKYSM, as identified by a broad stakeholder group ranging from government staff to residents and entrepreneurs, are detailed in Yukalang et al. [27]. This study is novel because it presents an overview of engagement with stakeholders from a wide range of levels in MSWM. This approach, while strongly recommended by UN-HABITAT in Solid Waste Management in the World’s Cities [2], is still often overlooked [6,28,29,30].

The aim of this paper is to present solutions based on an understanding of the region and its particular waste management challenges. The solutions for this study might be useful for other municipalities facing similar problems.

## 2. Materials and Methods

Following the Integrated Sustainable Waste Management (ISWM) framework, this study evaluated the current solid waste management system in TKY. The framework recognizes the importance of evaluating local conditions and needs and creating place-appropriate solutions [4]. 

**Figure 2:**
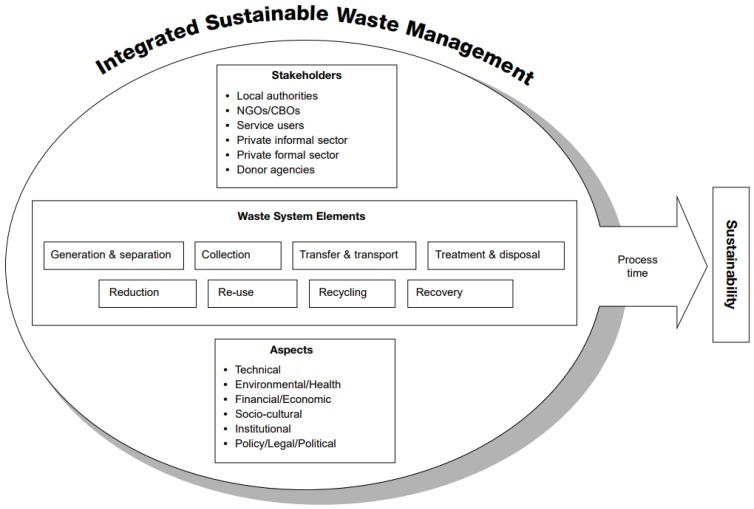
Integrated Sustainable Waste Management model (Source: Putting Integrated Sustainable Waste Management into Practice, 2004) [31].

ISWM theory recognizes three key components: the stakeholders affected by or engaged in waste management, physical or practical elements of the waste management system and an array of ‘aspects’ that directly affect waste management including political and cultural influences (Figure 2) [31].

To gain an understanding of the MSWM system, the waste management processes from point source to final disposal were observed, and primary and secondary reports and data scrutinised. 

In conjunction with site visits, observations, and secondary data analysis to determine the specific waste management issues facing the TKYSM, semi-structured, one-on-one interviews and focus groups were conducted to further investigate and understand the perspectives of local stakeholders [32].

Following the framework and criteria described by UN-HABITAT stakeholders were identified, including service providers, citizens, external agents or experts) [2]. Research instruments, for the focus groups and interviews were approved by the Flinders University Social and Behavioural Research Ethics Committee (Project Number 6784) in April 2015. Purposive sampling and snowball sampling (nonprobability sampling approach) [33,34,35,36] were used to select 34 in-depth one-on-one interviewees and 24 participants for three focus groups. All participants participated voluntarily [36]. Data collection was carried out between May 2015 and August 2016 in the TKYSM, Kantharawichai, Maha Sarakham Province.

In this study, participants were selected to represent a broad cross-section of Thai society across a range of different socio-economic groups, including low, medium and high status (Table 1). The defining characteristics of participants was employment and education (because they are related to income) [37]. Participants with tertiary education and in white collar employment or business ownership were classified as high socioeconomic status (SES). Those on low incomes doing more menial work were classified as low SES, this includes operational staff and waste pickers. Villagers and village leaders are mostly farmers, and although some of them have leadership positions in their villages, they do not receive a high income and were therefore classified as middle income. Students were also classified as medium because they have a high education, although low incomes. A number of different strategies were employed to identify, contact, or approach potential participants.

Purposive sampling was the main technique used to identify participants for the structured and non-structured interview [36]. Interviewees were selected by four different strategies. First, at community meetings a senior delegate introduced the research project and invited village leaders to participate. Second, the researcher created a listing of establishments (e.g., restaurants, markets and dormitories) from two urbanised zones and selected a sample from three different-sized businesses (small, medium and large). The researcher visited selected entrepreneurs and delivered the research instruments. Third, emails were sent to some known academics asking them to participate in the study. The email invitation encouraged these first contacts to forward the invitation to other relevant academics with expertise in waste management (snowball sampling). Finally, the researcher called secretaries of waste management administrators, inviting the participation of the senior staff. On deciding to participate, all invited participants contacted the researcher and made time for appointments. The waste operator for MSU was contacted as were the academics, and the recycling trader and scavenger. Unstructured interviews were conducted with the recycling trader, the operational staff within the university, and the waste picker. To do these, the researcher visited them at their workplace and the landfill site, and could interact with them onsite. The strategy used to select focus group participants was purposive sampling. Key contacts were asked to suggest possible participants who were then approached to ask whether they would be willing to participate. Residents from different villages, students (tenants) from different off-campus dormitories and waste management operation staff of TKYSM volunteered to participate in the focus groups. Three focus groups were run, with up to ten participants each (Table 1).

Key questions asked of interviewees and focus group participants are shown in Table 2 and Table 3.

Interviews took from 20 to 40 min. Focus groups took from 60 to 90 min. Conversations from both interviews and focus groups were recorded using digital audio recording devices. During the focus groups, the researcher moderated, and assistants took notes and managed the recording device.

Audio files were transcribed in Thai language onto a word processor and then uploaded to NVivo, qualitative data management software, for coding and analysis [38]. Quotes presented here are well articulated responses to questions and represent themes raised by multiple respondents. 

## 3. Results

The results presented here largely reflect respondents’ ideas for solutions to waste management challenges in TKY. These are preceded by a brief overview of the site visits and observations made by the researcher, which confirm the scale and impact of uncollected and unmanaged municipal waste. 

### 3.1. Observations

**Figure 3:**
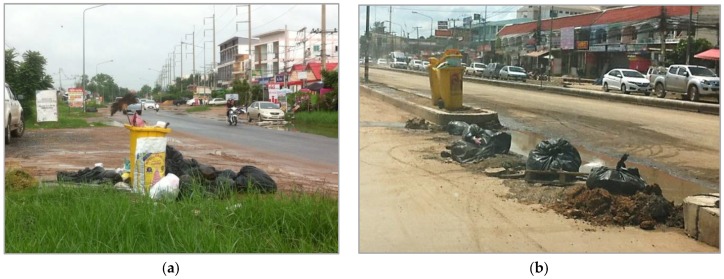
Typical scene of road-side waste in Tha Khon Yang. (**a**) Urbanized zone 1 (Observed 2/09/2015); (**b**) Urbanized Zone 2 (Observed 25/07/2016).

Site visits along the streets of TKY confirmed that pollution from open dumping is widespread and common. The photographs show that the population of TKY dispose of their waste onto curbsides in plastic bags, which break apart, and litter scatters into the streets and adjacent spaces. These temporary waste disposal sites are unsightly and generate strong odours (Figure 3).

### 3.2. Participants’ Responses

The majority of participants in this study agreed that solid waste management is a problem for TKY. The results suggest that socioeconomic status (SES) (defined here by education and employment) had very little influence on attitudes of respondents to waste management. All participants regardless of their SES were very concerned about waste management and gave detailed feedback about how to improve the MSWM system for TKY.

**Figure 4:**
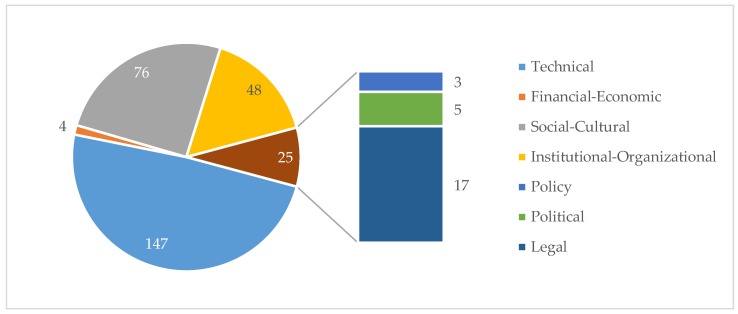
Thematic content of all of the quotes emerging from the unstructured and semi-structured interviews and the focus groups, categorised according to the categories of the ISWM (policy-legal-and political-based quotes are presented together in the pie chart and separated for clarity).

When asked, “what improvements need to be made in regard to the municipal solid waste management in TKY?”, the most common response across all stakeholder groups related to ineffective collection processes that lead to waste accumulation. In other words, there was an emphasis on the technical aspects of MSWM (Figure 4). Lesser consideration was given by participants to the institutional and organisational, social-cultural, legal and political, financial and environmental and health aspects of MSWM. All quotes presented here are coded from answers from the semi structured and unstructured interviews and focus groups.

The results presented below are organised according to the structure of the ISWM framework in Figure 2. The remainder of this section presents the recommendations of participants as to how to improve the MSWM system of TKY, arranged according to the Integrated Sustainable Waste Management (ISMW) framework.

### 3.3. Technical Recommendations

The technical components (Figure 2), provide a guideline by which to consider the practice of waste management. The technical components of an efficient waste system are two tiered. The first tier includes the process for collecting and transferring waste (facilities and equipment). The second tier corresponds to waste reduction techniques such as composting, recycling or reusing waste [4]. 

Technical solutions associated with MSWM were the primary focus of participants. Each of the three focus groups and 25 (81%) interviewees gave 147 individual quotes dedicated to technical solutions (Figure 4). Participants cited solutions about the need for facilities to support waste separation, waste containers, waste collection points and more or modified waste collection vehicles. Technical solutions also included increasing the frequency of waste collection days, and changes to collection routes. Second tier solutions of waste reduction through recycling and composting were also mentioned.

“*I am interested in waste disposal and sustainable management systems ... having a truck collecting waste every day, different types of waste bins and having junk shops buying recyclable items*.”

ID13 [In-depth interview]

A restaurateur suggested that changing packaging products was an efficient way to reduce waste entering the waste stream, but that this had knock-on effects.

“*Foam *[for packaging take-away food items]* poses the same problem as plastic, they are not bio-degradable. But they are cheaper than the bio-degradable products like [containers] made from bagasse *[sugarcane]*. If we want to use bagasse for food containers, we need to raise the price of the food we sell*.”

R6 [In-depth interview]

According to Figure 2, ‘waste generation and separation’ is the starting point of the waste management system. Many participants (three focus groups and 11 (35%) interviewees), thought that a simple and convenient waste separation system for the community was an important solution for waste management in TKYSM. The provision of coloured or labelled bins to assist waste separation would assist people to undertake waste separation at the source.

“*How can we *[off campus student accommodation owners]* help? First, we have to provide different kinds of bins with attractive signs, and then announce it to tenants. The tenants will be then able to understand which bin is for recycling, garbage or food waste*.”

D4 [In-depth interview]

Compartmentalised trucks allowing for waste separation at the collection point or multiple runs for different waste types (e.g., one run landfill, one run recyclables) were also suggested as mechanisms to assist in waste separation.

“*Waste collection trucks could have separate boxes for different types of waste.*”

F2 [Focus group]

“*The TKYSM needs to administer an appropriate waste separation system and process using separate waste collection trucks for different waste types; also, there needs to be waste separation bins for people*.”

ID11 [In-depth interview]

“*If people separate their waste then trucks could collect food waste one day; and another day collect paper or recyclable waste*.”

ID11 [In-depth interview]

A number of different ideas about ‘collection’ and ‘transfer and transport’ (Figure 2) were suggested by respondents including: clearly identifying waste collection points, improving the frequency, efficiency and effectiveness of waste collection; increasing the number of trucks and/or improving truck capacity or design. It was also noted that Geographic Information Systems (GIS) modelling could help improve the efficient design of waste collection routes.

“*Effective waste collection routes will save time and could expand the system to collect waste in every area*.”

D4 [In-depth interview]

‘Waste treatment and disposal’ is the final element in the waste management system (Figure 2). Currently, TKYM has limited facilities to process its own MSW. 

In terms of tier two solutions to reduce waste, participants identified the need and potential for the construction of a waste separation facility, recycling centres and/or community or household composting facilities. Several participants and entrepreneurs (from restaurants) saw the benefit of changing organic waste to compost and utilising food waste to feed animals. This would result in a reduction of waste being processed through the system and lower the volume of waste going to landfill.

“*TKYSM should establish a recycling center for buying recyclable waste*.”

ID03 [In-depth interview]

“*Some communities might have a small composting plant. TKYSM could encourage the villagers to make composting on site for each household*.”

ID11 [In-depth interview]

According to a staff member from the TKYSM, the municipality plans to develop a pilot waste separation and composting project in two villages. Each village will have their own waste separating site and local people will be hired sort the waste. This respondent concluded that setting up MSWM programs and encouraging local people to separate waste in rural areas would be easier than in the urban areas where space is limited. If this pilot project is successful, it could be used as a model to apply in other villages.

“*I would start the pilot project with two rural villages. Rural communities will have black bins for organic waste and other colored bins for general waste. People could learn to manage and separating waste by themselves*.”

ID05 [In-depth interview]

A few participants (4 (13%) interviewees) suggested the Provincial government should identify and develop a new landfill site specifically for the TKY.

“*Find space and develop a landfill site for TKY*.”

ID06 [In-depth interview]

### 3.4. Financial-Economic Recommendations

The technical solutions described above require funding. Two academics and one external expert stressed the importance of a suitable budget for further development of the system. The ‘financial/economic’ aspect of Figure 2 refers to costs inherent in the operation of a waste management system as well as sources of revenue including fees and income generated from the sale of recyclable items. 

A number of ideas were offered by study participants to increase the funds available for advancing waste collection and management.

“*Why don’t they *[the municipality]* collect fees via taxes *[like]* organizations such as water, electricity or other services*?”

ID11 [In-depth interview]

Collection of fees from households towards their waste management in TKYSM is ad hoc (see Yukalang et al. [27] for details). A solution suggested to improve revenue raising was to give the community more power to manage their own waste collection fees.

Another participant noted that money from selling recyclable waste could be collected and funnelled back into MSWM―perhaps via a village waste fund. The potential value of waste, through recycling and reuse, was noted by a number of participants.

“*We have to see value in waste; it is a resource; *[scavenging]* is the kind of work that can earn money. In a municipality, if someone knows how to manage *[sale of recyclables]* well, that is money. …However, to have this, *[appropriate management of MSW]*, available space and a proper budget is necessary*.”

ID10 [In-depth interview]

“*Waste from the newer *[wealthier]* communities can make more money-people there dispose of larger quantities of valuable recyclable waste. On the other hand, people in older villages usually separate recyclable waste to sell it anyway*.”

ID05 [In-depth interview]

### 3.5. Social and Cultural Recommendations

Participants identified ‘socio-cultural’ aspects of MSWM in terms of raising public awareness and enhancing participation in waste management. Socio-cultural aspects were the second most commonly cited solutions. Social and cultural solutions were generated by the three focus groups and 21 (68%) interviewees, resulting in a total of 76 individual quotes (Figure 4).

When asked, “who should take responsibility for waste management?” most participants replied that ‘everyone’ should take responsibility. In order for improved practices and changes in behaviour, participants explained that a change attitude of TKY citizens to waste and its management is essential. Solutions for changing attitudes included increased education, making use of important cultural places, implementing rewards and increasing social pressure to do ‘the right thing’.

“*We need to educate people to think that waste can be precious things to change their attitudes*.”

ID01 [In-depth interview]

“*It takes time to install or use social pressure to make people know that it is wrong to dispose of waste … if someone knows how to manage it well, that is a resource and money*.”

ID10 [In-depth interview]

With the exception of operational staff and market owners, every stakeholder group suggested that education is the mechanism by which to change attitudes. There is potential for the Thai education system as a whole to help usher in change, but other key leaders and influential people were also identified as playing a role.

“*A person that can reach people such as village health volunteers and staff from primary health care centres could educate people. Leaders of villages could inform people using basic knowledge*.”

F2 [Focus group]

“*The [national] Education Department could set waste management as a national issue; with every school separating its waste*.”

ID08 [In-depth interview]

Some participants were already involved in waste management education, and others (village leaders, school teachers and academics) indicated a willingness to be involved in the future.

“*Now, I *[school teacher]* am starting to train students about waste separation. There will be discussion in the classrooms. I will focus on the students by building discipline in them*.”

ID15 [In-depth interview]

“*As I am a leader of a village, I have a special responsibility for waste management by informing local residents to separate their waste.*”

ID02 [In-depth interview]

Some villages’ host training programs (such as composting, biogas fermentation and establishing recycle banks where waste materials are bought and sold) run by municipality staff and academics from Mahasarakham University. Through these projects, people learn the value of waste, and as a result, reuse, recycle and reduce waste going to landfill. 

Thailand is going through a transitional period where the culture of older Thai people (particularly in city areas) is quite different to younger generations. Several university students said that education about waste management needs to have impact.

“*To inform people about waste, it needs to be something interesting. What about a short film contest? This would ensure that information can easily reach and inspire students to be concerned about waste*.”

F2 [Focus group]

A restaurateur (and a dormitory owner) highlighted that temples are an important hub for the people of TKY. Ninety-five percent of Thais are Buddhist [39]. Temples provide a meeting place for the community and as such can provide a place for learning.

“*I used to write waste management songs for singing in a temple*.”

D5 [In-depth interview]

Temples receive recyclable waste as donations and the monks sell on these goods. A participant suggested that this practice could be encouraged and expanded. As the monks are respected it is likely citizens would bring high quality recyclable items.

“*The TKYSM should ask a temple to be a recycling center. People will bring good recyclable waste there*.”

R2 [In-depth interview]

Some participants suggested financial rewards and praise as methods as encourage people to participate in waste management.

“*How about giving a reward for people who separate waste? If we give recyclable waste to the owner of accommodation and in return, they reduce the electricity cost for us, it would be nice*.”

F2 [Focus group]

“*We can also promote it as beneficial for student accommodation to get a five-star award. The benefit of sorting waste is earning money back and it is also easier to manage by making rules for tenants.*”

ID09 [In-depth interview]

### 3.6. Institutional/Organisational Recommendations

Solutions for the ‘institutional/organisational’ aspects (Figure 2) are focused on the TKYSM, around organisational structure, planning and decision-making, and staff capacity for managing waste. Two focus groups and 19 (61%) interviewees mentioned institutional or organisational solutions, with a total of 48 quotes focussed largely upon improving municipal staffing matters (Figure 4). For example, staff duties are often split across portfolios and waste management is not always the priority; employees need to have a better understanding of their roles and responsibilities; staff capacity needs to be improved through training or education programs; and there are too few staff. 

Participants identified the need for defined duties within the TKYSM. A mechanism is also required to ensure that individual staff fully undertake their specified role:

“*They* [the municipality should] *require staff to work seriously.*”

ID10 [In-depth interview]

The municipality would benefit from a strong and clear MSWM implementation plan to guide staff at all levels, from the director to the collectors, including a clear mandate about staff duties and responsibilities. It was also suggested that staff responsible for MSWM should not have other responsibilities that take them away from their primary role. 

Developing staff capacity will improve MSWM. It may be that the TKYSM needs to invest in staff training. One expert suggested:

“*Private consultants may need to be engaged to train staff appropriately*.”

ID10 [In-depth interview]

According to some participants, TKYSM is not entirely responsible for finding solutions for its waste management problems. One focus group and 14 (41%) interviewees indicated that stronger relationships with higher tiers of government and with other organisations is important. For example, there is potential for TKYSM to seek support and advice from the Mahasarakham University which has implemented a good waste management system. The University could be a model of MSWM efficiency and provide expertise to design improved system operations, such as more efficient waste collection routes and to build municipal staff capacity.

“*The Mahasarakham University can design and offer new systems for the TKYSM. I do understand that the TKYSM has a limited budget, [so, for example] if the university helps by giving some suggestions about effective waste collection routes to suit a budget, this should include staff requirements and routes required per day*.”

D4 [In-depth interview]

In addition to TKYSM’s responsibilities, participants indicated that they thought that higher tiers of government should play a role in MSWM because they have a responsibility to assist subdistricts to work and plan together. An example of how institutional cooperation might help was suggested by the director of the TYKSM, who referred to establishing a new landfill site.

“*We need help from the Provincial Administrative Organisation and the District Office. They have power to find space and develop a landfill site. They have more authority to create cooperation between sub-district municipalities. How can this happen*?”

ID06 [In-depth interview]

### 3.7. Policy, Legal and Political Recommendations

‘Policy, legal and political’ aspects are those supporting conditions that help regulate the proper management of waste. Twenty-two opinions from one focus group and 7 (23%) interviewees indicated that legal and political factors influenced MSWM and that laws and regulatory frameworks should be strengthened.

“*If they *[TKYSM]* don’t hurry up and develop municipality law, waste will be difficult to manage and control. The best solution is to let people follow the law. Law is important; it can do everything. People love laws*.”

ID08 [In-depth interview]

“*Actually, the law *[the Public Health Act A.C. 1992]* under Section 3: Waste and sewage disposal, the municipality has the authority to dispose of waste in an authorized area. So, they need to set rules for waste management. They need to consult lawyers from the Public Health lawyer center for establishing the municipality law. And [TYKSM] has to follow it seriously and continually*.”

ID10 [In-depth interview]

One participant commented on the potential for policy in provincial and sub-district level to bring credit to the area:

“*Making a provincial policy might be a remarkable campaign for this province*.”

R4 [In-depth interview]

Several academics gave suggestions to push politicians to engage with MSWM projects, including:

“*We need to encourage them *[politicians]* as it will help them get votes. Don’t talk about environment. Because the politicians will care only about their [re-] election … we can say ’‘If you can keep the town clean, within the next 10 years, people will vote for you*.’ ”

ID10 [In-depth interview]

Figure 4 presents a summary of the focus of all of the quotes emerging from the unstructured and semi-structured interviews and the focus groups, categorised according to the categories of the ISWM.

Measurable, sustainable indicators are presented in Box 1.

Box 1 **Measurable sustainable indicators for MSWM in TKYSM.**

Measurable sustainable indicators for MSWM in TKYSM (adapted from the ISWM framework for six target aspects) could include:

Technical aspects:

- Volume of waste going to landfill is decreased within a short and long term.
- Facilities for waste separation system are established, including waste containers and waste collection points.
- A waste separation plant, recycling centre and composting plants are established.

Financial-economical aspects:

- Cost of waste disposal to landfill site is reduced compared with previous years.
- Income is generated from selling recyclable items.
- Locals or scavengers are hired in the new waste management system.
- Implementation of a waste collection fee-paying system that easy to understand and complied with.

Social and cultural aspects:

- Engagement with, and support from, the community in MSWM (measured by survey).
- Development of a waste management team or network for monitoring, which consists of local residents, entrepreneurs, students with help of TKYSM, schools and university.

Institutional/Organisational aspects:

- Production of a well-developed, articulated MSWM strategy and implementation plan.
- Hiring of adequate staff and implementation of training courses for MSWM.

Policy, legal and political aspects:

- Production of well developed, articulated MSWM policy, municipality laws and associated rules.

Environment/Health aspects:

- Implementation of an environmental health assessment and monitoring system (longer term).
- A decrease in the number of people complaining about the MSWM.

## 4. Discussion

Observations indicate that the MSW in TKY is not being managed appropriately. Tha Kong Yang has experienced rapid population and economic growth. It is becoming more affluent [21,40]. This growth and prosperity have led to an increase in the use of consumer items which has resulted in a substantial increase in the production and volume of solid waste. This presents the difficult task of dealing with unmanaged and accumulated waste that is causing environmental, health and aesthetic problems. In TKY there is pressure on local government to improve its existing waste management program [21,27,40].

This study aimed to identify viable solutions to TKY’s MSW problems that may potentially provide guidance to other localities in developing countries also facing waste problems resulting from rapid change. To do this, the ISWM evaluation framework proposed by US EPA (2002) [4] was used to structure the evaluation and help identify solutions to both waste accumulation and waste management. It is well established that successful changes to waste management require an understanding and consideration of local context [2,41]. Therefore, extensive community consultation and engagement with experts was undertaken to develop an understanding of the region and its waste issues. Study participants identified an array of different strategies and solutions to respond to the equally diverse set of problems associated with waste management. This discussion provides an overview of the strategies and ideas (from structural and procedural to practical actions) towards an improved system and a reduction of unmanaged solid waste currently accumulating in the municipality.

There are many opportunities to improve local infrastructure and operational capacity around solid waste management. There is recognition that the current system cannot be sustained, with its focus on landfill as the current sink for waste [40]. Waste management strategies need to differentiate between rural and urban areas. Strategies emerging from this study are not necessarily highly technical or complicated and as such may be implemented with some careful thought and good planning. It is important that appropriate technologies are developed for and implemented in developing counties. There is compelling evidence that it is ineffective to transpose complicated or expensive technology designed for developed countries to developing countries [12,13,41].

Presented below in order of importance and urgency are key solutions. Aspects demanding immediate attention are presented first, followed by longer-term targets. Key findings are based on the outcomes of this study. The key solutions are: development of a municipal waste management policy and an associated implementation plan; reduce the need for the landfill by generating a waste separation program (including education, infrastructure and economic policy), improving the existing waste collection system, and improving the financing of waste management.

### 4.1. Key Solution 1: Develop a Locally Relevant Waste Management Policy and Implementation Plan

The TKYSM does not yet have a strategic vision or associated policy for waste management. Clear policy for waste management is needed to address both immediate and long-term goals. Daichai et al. [42] (p. 1) noted, when referring to another municipality in Thailand, that “the municipality has to set a clear policy goal of municipal waste management, short-term, and long-term action plans.”

Overarching policy should be developed in conjunction with an implementation plan. The US EPA “Solid waste management: A local challenge with global impacts” notes that when it comes to ISWM, government plays an important role in planning, developing, and managing day-to-day operation of solid waste management activities [4]. The TKYSM has a duty and the power to manage is own waste management system [43,44], and is therefore responsible for developing both policy and plans. However, as observed by Amornvivat (2004), many local administrations around Thailand are poorly prepared to take on these responsibilities, as many of them are “considerably too small with regard to mandatory services” [45] (p. 18). This includes problems with “efficiency of service deliveries, absorptive capacity, local autonomy, and financial adequacy” [45] (p. 3). Direction could be taken from the Thai Government’s Roadmap [16], and local policy be developed in conjunction with partners such as academics from the Mahasarakham University, who can provide both legal and technical expertise [3]. A workable implementation plan to address the MSWM problem can be based on the key findings outlined below. 

### 4.2. Key Solution 2: Reduce the Volume of Waste Going to Landfill by Establishing a Waste Separation System

The Thai Government’s Roadmap states, “communities and municipal authorities are encouraged to reduce waste, implement waste sorting at source and dispose of waste in an appropriate manner” [17] (p. 14). There is an urgent need to reduce the amount of waste going to landfill from TKY. The landfill site currently used by TKYSM is costly and not sustainable in the long term (TKYSM currently pays the Mahsarakham provincial municipality to leave waste at this landfill. It is estimated to be more than half of the TKYSM’s budget). Establishing a new landfill site is a complex, expensive process and likely to be opposed publicly [46]; isolating a suitable site will be challenging [11], as land close to the TKYSM is prone to flooding [40].

A cheaper, more sustainable option would be to reduce the amount of waste going to landfill. Respondents in this study identified enthusiasm for such an initiative.

It is estimated that in TKY, organic waste (primarily food waste) contributes 60% of waste volume, and recyclable waste (resalable waste products including cardboard, paper, plastics and metals) almost 40% [21,25]. Therefore, waste sorting and separation and the diversion of organic waste and recyclables will almost eliminate the need for landfill dumping.

There are two possible approaches to waste separation. Separation can be accomplished at the source, then collected and taken away for reuse or recycling; or, unsorted waste can be collected and taken to a waste separation site facility for sorting. An administrator of the TKYSM and other respondents in the study prefer the second option. However, finding space to site such a facility will be the first hurdle. TKYSM residents are opposed to siting a waste separation facility close to the municipality due to concerns over environmental and aesthetic impacts [27]. 

**Figure 5:**
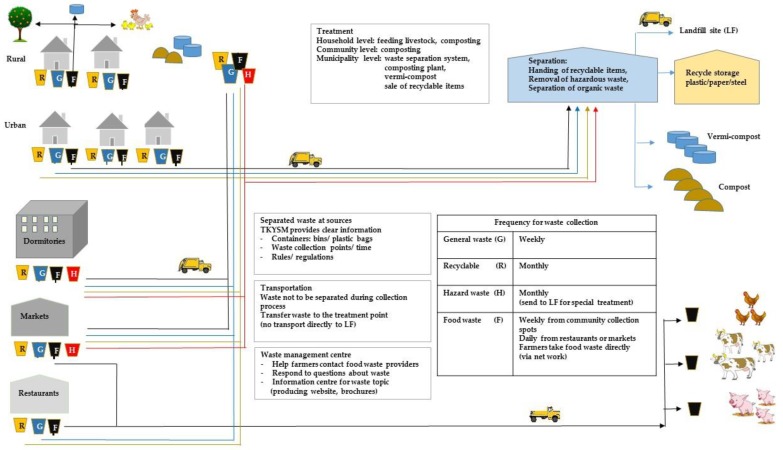
A schematic approach to waste separation components.

Perhaps a more acceptable approach will be to separate organic waste at the source and for it to be transferred directly to end users. However, organic wastes from rural and urban areas will need to be approached differently (as illustrated in Figure 5). Smaller facilities for sorting non-organic recyclable waste might be accommodated throughout the municipality. 

Should a coloured bin system be implemented to encourage waste separation, it would need a companion public information roll-out (Figure 5). Tai et al. [47] indicated that at-source separation was significantly improved when accompanied by multimedia advertorials (radio, television, newspapers and the Internet). This contrasts with Nixon and Saphores [48], who found that face-to-face communication between friends or colleagues was the most effective method of encouraging people to recycle. 

In TKY waste accumulation is seasonal. It varies according to the University timetable (e.g., it reduces during semester breaks when students vacate), to special events, and with farming calendars. TYKSM should also include in any new plans contingencies for emergencies, such as during floods, or landfill closure.

### 4.3. Key Solution 3: The Need to Initiate a Collection Service That Supports Waste Separation at Source

Facilitating waste separation at the source has the potential to drastically reduce landfill requirements. Viable suggestions for encouraging at-source waste separation included truck modifications (creating separate compartments within collection trucks to receive and segregate different types of waste). The potentially more efficient suggestion is to collect different types of waste on different days (Figure 5). The advantage of this second approach is the use of existing trucks rather than requiring investment in expensive modification or extra collection trucks. Food waste from restaurants could be delivered directly to farmers. In this scenario, the municipality may have a role to pay in providing a small truck to transport food waste (Figure 5). The use of restaurant food-waste as livestock feed has been successful in other countries either directly [49], or after processing through fermentation [50] or dehydration [51]. Given the added expense of processing food waste, it is suggested that direct transfer to farmers is the preferred approach. 

Collection routes need to be optimised to assist TKYSM meet collection schedules and to reduce costs. Collection and transport of waste is generally the most expensive aspect of MSWM. Das and Bhattacharyya (2015) have shown that route optimisation could reduce the collection path length by more than 30% [52]. This is supported by the work of Son and Louati [53] who modelled collection scenarios using GIS to and substantially reduced collection paths. TKYSM could engage with the Mahasarakham University to apply modelling technology to improve local collection routes. 

### 4.4. Key Solution 4: The Need for Support and Education of the Waste Producers

Improved citizen behaviours such as waste separation at source and following collection schedules requires their engagement and commitment. Respondents in this study perceived a lack of engagement resulting from disinterest in the environment [27]. It is clear from the literature that at-source waste separation only works if the necessary infrastructure is provided [48], and if the system is convenient and readily understood [54,55,56].

A common reason given as to why citizens of TKYSM did not separate their waste was the inconvenience of the task (e.g., having no time or space for recycling) [27]. This is supported by other research which showed that space for storage or distance from recycling centres results in reduced recycling behaviour [48]. Therefore, TKYSM should make separation facilities accessible (Figure 5). Making available the necessary infrastructure to undertake waste separation will be essential [56]. 

It is also important that residents understand what is required of them and that they are engaged in the process [3]. To date, efforts to change behaviour have experienced limited success for the municipality, the owners off-campus student accommodation and entrepreneurs [27]. A variety of communication tools tailored to end-users’ needs and level of understanding is essential [55]. The municipality will need to educate its citizens about any changes to the collection system and service (such as the introduction of different-coloured bins or community collection points).

Engaging citizens in the development of waste policies and planning may increase engagement in the uptake of new strategies [57].

To reduce food waste entering the waste stream participants in this study suggested that the TKYSM should arrange training for households or businesses about how to compost so as to encourage the practice.

Awarding good waste management behaviour as a strategy to encourage people to engage in waste separation activities was an idea proposed by students and some experts. TKY has more than 250 off-campus student accommodation facilities which are proving to be points of pollution [58]. Awards could include a recycling star-rating for dormitories, or fee reduction for tenants. The use of rewards―incentives as regulatory instruments―has been assessed in a number of evaluations including Garbosky (1995) [59], and Wilson and Balkau (1990) [60]. The use of ‘carrots and sticks’ to manage waste needs to be thoroughly investigated to ensure that the means justifies the required end. 

Participation in separation of recyclables is likely to be more successful in low income communities who can generate income from the sale of recyclables through buy-back centres or waste banks. Around Thailand, waste bank projects have been successful in schools, communities and universities [61,62]. A challenge might be to encourage wealthy communities or big businesses to undertake proper waste separation of recyclables. A reduced waste collection fee resulting from reduction of waste going to landfill might be a mechanism to ensure uptake by higher socio-economic communities or businesses [11].

TKYSM would benefit from the help of external agencies, primarily the Mahasarakham University, seeking assistance from experts in the areas outlined above. The TKYSM directors indicated that local people would prefer to listen to the opinions and guidance of new people, possibly because it may make the subject matter more interesting or because there is a belief in the expertise offered by University staff. Therefore, support from the Mahasarakham University would be very helpful in many areas, including the development of a communication strategy to inform people about new waste management plans and systems. The benefit of engaging with the local University is that being situated adjacent to TKYSM staff will understand the local context. 

There are currently public health volunteers living in TKYSM. These volunteers usually work with the primary health care centre (a local government organisation that is separate from TKYSM), sharing health information with villagers. Having already established relationships with villages, these volunteers could provide a conduit between the TKYSM and village communities. 

A communication breakdown between the municipality and wider community exists. The community feels uninformed and complained about methods of communication as being inadequate [27]. To ensure better communication between residents and the municipality, local waste management teams or centers could be established, overseen by the TYKSM, but staffed by villagers. Such centers may help establish a mutual understanding of MSWM between citizens and the municipality. Any planning for MSWM in TKYSM should engage the community. There have been many recommendations from participants about improvements to communication between the TKYSM and people in the community. These methods include written information (letters or brochures), online technologies (mobile device applications), and visual media (short films), all of which would guarantee widespread distribution of information. 

### 4.5. Key Solution 5: The Need for Support and Education of the TKYSM Staff 

There were a number of staffing related suggestions. Respondents thought waste management would be more effective if more TKYSM staff were employed. In addition, a clearer staffing structure and identification of roles and responsibilities need to be developed. TKYSM should prepare appropriate staff training for MSWM.

The technical skills of personnel employed by municipal governments significantly influence waste management systems [63]. It is one of many factors that influence waste collection and transportation waste [64]. Waste pickers may potentially staff recycling centres as they already use separation techniques seeking out high value recyclable items [65]. Waste scavengers have been successfully incorporated as part of recycling programs in other countries, including. Brazil, Colombia, India, South Africa [66], Nigeria [67], Tanzania [68], Indonesia [69], and China [70]. 

It was clear from site visits that the TYKSM needs to develop a system for monitoring waste. For example, records of waste volume are missing data for some days, and some years the waste volume reports are missing. Therefore, changes to the volume of waste overtime and changes to the waste stream are incomplete or unavailable. Easy-to-use, reliable monitoring [41] and recording systems are imperative to support long-term decision making in MSWM. 

### 4.6. Key Solution 6: Financial Considerations

The proposed new MSWM system will require a sufficient budget. The TKYSM should consider several ways to enhance its budget. An immediate step would be to introduce (and strictly adhere to levies) for residents and commercial enterprises in receipt of waste collection services. Second, the implementation of a functioning waste separation system, focused on recycling and composting, will generate an income from the sale of such products [71]. Simultaneously with a waste separation scheme is the need to introduce appropriate infrastructure to assist households. Residents could be required to buy their coloured waste bins from the TKYSM or use the ‘prepaid bag’ system that has been successful in South Korea [72]. Third, TKYSM spends more than half of its MSWM budget on disposal of waste to landfill. Diversion of waste away from landfill will result in substantial savings. Finally, the TKYSM could research ways to reduce total expenditure for waste management, such as waste-to-energy production.

### 4.7. Key Solution 7: Cultural Considerations—Engaging the Use of Temples

Most Thais follow the Buddhist faith, and as such, their temples are important social and cultural meeting places [39]. They are also places for contemplation and learning. Quality recyclables are already brought to the temples as donations. As temples are kept clean, they could potentially provide sites for recycling. Engaging with monks may provide an important strategy for MSWM, particularly in Thailand.

### 4.8. Unanticipated Outcomes

Despite the Thai Roadmap’s focus on waste reduction [17] this was rarely mentioned by study participants as a strategy for solving MSWM in TKYSM. One person (a mini-mart owner) mentioned that he asks customers whether they need a plastic bag in a bid to reduce bag use. One of the contributing factors to the MSWM problem in TKY is rising affluence and the associated increase in consumer goods [40]. Waste reduction is an obvious and much-cited solution to waste management; however, most MSWM plans focus on reuse and recycling [73]. The introduction of fees for waste collection and disposal has seen reductions in waste generation [74].

The ISWM framework (Figure 2) has a ‘health and environment’ aspect. Participants in this study did not offer solutions about how to better protect the environment and human health from MSW. This was despite many comments about such problems. It is noted by the authors that the key findings listed above are in themselves inherent solutions to environmental and health problems. 

## 5. Conclusions

This study has identified simple, logical solutions to both the waste accumulation and waste management problems in TKYSM. This was achieved by engaging with the MSW stakeholders, including waste generators, staff from the TKYSM, academics and administrators. 

Pressure to establish a successful MSWM system in TKY is increasing due to costs associated with the current approach of taking waste to a landfill outside of the TKYSM area, which is expensive and not sustainable. To achieve effective MSWM in TKY, establishing technical and expensive solutions are not recommended. Establishing a new waste disposal site is not a feasible option because it is not sustainable nor suitable. Instead, a simple system based around recycling and reusing is proposed. The primary component of the plan requires appropriate waste separation, which takes into account the lifestyles of residents in urban, rural and commercial areas. The focus should be on food waste and recyclable materials, which together comprise almost all of the waste stream. Appropriate separation containers must be provided, and regular collections initiated. The management facilities that is readily understood by the residents and businesses and also by the operational staff involved in MSWM. 

Ensuring of awareness and uptake of waste separation by local citizens is a significant challenge for the local municipality. Information must be provided across the public domain in a way that is direct and reduces confusion while increasing awareness among local citizens.

Rules and regulations have to be clear and be developed with a bottom up approach to ensure that the changes match cultural and distinct local needs in the area. This also includes the revenue collection processes. 

Overall, a common theme has emerged that shows input from the local population into the development of the MSWM system will be vital to its success of the project. This target needs significant cultural shift in government policy and human behaviour, including the way people think about waste, which will assist in the development and implementation of robust MSWM systems for TKY. 

### Further Research

Establishing an appropriate monitoring system to determine the types, volumes and seasonality of waste production in TKY will guide future research. Engagement between the TKYSM and the Mahasarakham University will provide opportunities for further research and evaluation. Other potential research areas include an evaluation (cost, benefits and outcomes) of the new waste separation system in the TKYSM to inform decision making, applying GIS to determine better waste collection routes for the TKYSM, assessing the level of community engagement and waste management in the TKYSM, establishing a coaching and mentoring program for the waste management team, undertaking an Environmental and Health Impact assessment in the waste management system in TKY and research into the application of this model in other rapidly urbanizing areas in Thailand.

## Figures and Tables

**Table 1:** In-depth interviews and focus group participants.

List of Participants	Number of Participants		Socioeconomic Status *
	In-Depth Interview	Focus Group	
**Waste Management Service Providers**			
Administrators of the TKYSM	3		High
Operational waste management staff of the TKYSM		10	Low
**Waste Management Service Users**			
Leaders of villages	4		Medium
Restaurateurs	6		High
Off campus student accommodation owners	5		High
Minimart Owners	4		High
Local residents living in Tha Khon Yang area		8	Medium
University Students, living in off campus student accommodations in Tha Khon Yang area		6	Medium
**External agents and experts**			
*Academics*			High
University lecturers of Mahasarakham University	3		High
University lecturers of Mahidol University, Bangkok	3		High
School teacher from Primary School, Tha Khon Yang Sub-district, Maha Sarakham Province	1		Medium
*Other organisations related to MSWM*			
Director of the Provincial Natural Resources and Environment Office, Maha Sarakham Province	1		High
Waste operator of Maha Sarakham Town Municipality	1		High
Waste operator of Mahasarakham University ^1^	1		High
Recycling trader ^1^	1		Medium
Scavenger in Landfill site of Masasarakham Town Municipality ^1^	1		Low
Total	34	24	

^1^ An unstructured interview was used for adding extra information and to avoid bias. * See description in text.

**Table 2:** Key questions for interviews (note: these interviews were semi-structured, and 3 unstructured interviews were also conducted).

Number	Key Questions for Interviews
1	How effective do you think the municipal solid waste management is in the TKY (a scale of 1–5 where 1 is very ineffective and 5 is very effective)?
2	What are the most successful or best aspects of solid waste management in TKY?
3	In your opinion, what are the main challenges or worst aspects of solid waste management in this area?
4	What are the causes of, or obstacles for, municipal solid waste problems in this area?
5	What improvements need to be made in regard to MSWM in the TKY?
6	Do you think the waste problems affect (a) the environment (b) health of people (c) operational costs of waste management (d) other aspects in this area? (these were asked as four separate questions)
7	What technologies do you think are needed to improve solid waste management in the TKY?
8	What improvements could be made to the MSWM system?
9	What is the most important aspect that should be addressed?
10	What improvements could be made to help you undertake your own role (in the MSWM sector) more effective?
11	Who should be responsible for making these changes?

**Table 3:** Key questions for focus groups.

Number	Key Questions for Focus Groups
1	What are the most successful or best aspects of solid waste management in TKY?
2	In your opinion, what are the main challenges or worst aspects of solid waste management in this area?
3	What are the causes of, or obstacles for municipal solid waste problems in this area?
4	What improvements need to be made to overcome these problems?
5	What is the most important aspect that should be addressed?
6	What is the first aspect that you think it could be done as soon as possible?
7	Who should be responsible for making these changes?
